# Cranial bone flap resorption—pathological features and their implications for clinical treatment

**DOI:** 10.1007/s10143-020-01417-w

**Published:** 2020-10-12

**Authors:** Jennifer Göttsche, Klaus C. Mende, Anastasia Schram, Manfred Westphal, Michael Amling, Jan Regelsberger, Thomas Sauvigny, Michael Hahn

**Affiliations:** 1 Department of Neurosurgery, University Medical Center Hamburg-Eppendorf, Martinistrasse 52, 20246 Hamburg, Germany; 2 Institute for Osteology and Biomechanics IOBM, Hamburg University Medical Center, Lottestrasse 59, 20246 Hamburg, Germany

**Keywords:** Cranioplasty, Autologous, Failed, Bone flap resorption

## Abstract

Cranioplasty following decompressive craniectomy (DC) has a primary complication when using the autologous bone: aseptic bone resorption (ABR). So far, risk factors such as age, number of fragments, and hydrocephalus have been identified but a thorough understanding of the underlying pathophysiology is still missing. The aim of this osteopathological investigation was to gain a better understanding of the underlying processes. Clinical data of patients who underwent surgical revision due to ABR was collected. Demographics, the time interval between craniectomy and cranioplasty, and endocrine serum parameters affecting bone metabolism were collected. Removed specimens underwent qualitative and quantitative histological examination. Two grafts without ABR were examined as controls. Compared to the controls, the typical layering of the cortical and cancellous bone was largely eliminated in the grafts. Histological investigations revealed the coexistence of osteolytic and osteoblastic activity within the necrosis. Bone appositions were distributed over the entire graft area. Remaining marrow spaces were predominantly fibrotic or necrotic. In areas with marrow cavity fibrosis, hardly any new bone tissue was found in the adjacent bone, while there were increased signs of osteoclastic resorption. Insufficient reintegration of the flap may be due to residual fatty bone marrow contained in the bone flap which seems to act as a barrier for osteogenesis. This may obstruct the reorganization of the bone structure, inducing aseptic bone necrosis. Following a path already taken in orthopedic surgery, thorough lavage of the implant to remove the bone marrow may be a possibility, but will need further investigation.

## Introduction

Cranioplastic surgery following decompressive craniectomy (DC) is a well-established neurosurgical intervention. The operation not only restores the integrity of the skull but also seems to have positive effects on cerebral blood flow and cerebrovascular reserve capacity [12, 16, 41]. There is evidence that the rehabilitation potential as well as the neurological outcome of patients improves after cranioplasty. In addition, the procedure helps to achieve a cosmetically satisfactory result [4].

The restoration of the integrity of the skull can be achieved either by reimplantation of the autologous bone or by allogenic material such as bone cement (polymethyl methacrylate (PMMA)) or a previously manufactured patient-specific implant (PSI) made of titanium or polyether ether ketone (PEEK).

The literature regarding a potential correlation between graft material and infection rate is inconsistent [1, 3, 17, 19, 20, 22, 24, 38, 42]. Matsuno et al. describe a significant infection rate for titanium mesh compared to autologous bone flaps and PMMA [26]. However, while the implantation of the autologous bone is associated with significantly lower costs than the manufacturing of a PSI, a particular disadvantage remains: the occurrence of aseptic bone resorption (ABR) is a specific and not to be underestimated complication of this method that leads to a considerable number of patients requiring further surgery [30, 39].

This resorption leads to structural instability and consecutive loss of protective function in addition to the already existing risk of infection [8].

The rate of ABR varies in the literature with incidences around 20% [11, 39]. Bone flap fragmentation, shunt-dependent hydrocephalus, and especially, young age have been discussed lately as risk factors leading to ABR [5, 11, 13, 14, 25, 28]. Furthermore, some authors suggest a relationship between the method of storage, preparation of the implant, time interval between DC and reimplantation (“freezer time”), or the size of the defect [9, 18, 31, 33, 43]. A possible contamination and subsequent low-grade infection with *Propionibacterium acnes* has been discussed as a potential risk factor as well [6, 37]. Schütz et al. were further able to show that hypertensive patients treated with ACE inhibitors had a lower ABR rate than patients treated with other antihypertensive medication or patients who do not suffer from arterial hypertension [35].

Despite these frequent descriptions of ABR in the literature, we still lack a thorough understanding of the physiology of bony reintegration at the skull and thus the underlying pathology leading to the pathological process of resorption.

Therefore, the aim of this work was the histopathological examination of resorbed and intact bone flaps to gain a better and detailed understanding of the underlying processes.

## Materials and methods

Eleven patients suffering from aseptic bone resorption leading to a revision surgery after initial reimplantation of their autologous bone flap between 12/2013 and 02/2017 were included in this monocentric cohort study. In addition, two controls were examined. These were also obtained from patients who had undergone hemicraniectomy and were cryopreserved postoperatively. Since these patients had died before reimplantation was possible, these implants were used as controls.

This study was conducted according to the Declaration of Helsinki, local and institutional laws, and was reported to the local ethical committee (No. WF-093/20).

### Clinical data

**Fig. 1:**
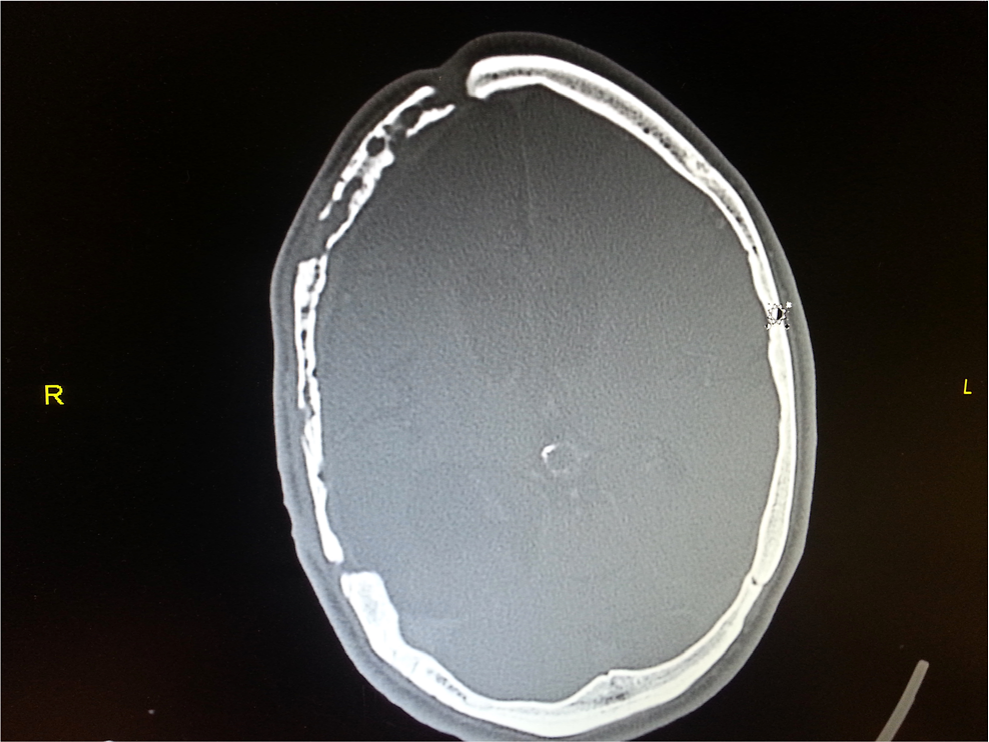
Bone flap resorption on CT scan

Aseptic bone resorption was ascertained via multimodal diagnostics including anamnesis, examination, and CT imaging of the skull (Fig. 1). In our clinic, all patients who have undergone surgery are routinely invited to our consultation hours for a clinical follow-up examination. A CT scan was only performed if there was a clinical indication of aseptic bone resorption, e.g., skin atrophy or a defect.

Patient data including demographics, primary diagnosis leading to DC, defect size, and time interval between craniectomy and cranioplasty were collected. Additionally, endocrine serum parameters affecting bone metabolism including osteocalcin, calcitriol, calcifediol, bone alkaline phosphatase, parathyroid hormone, calcium, and thyroid levels were collected.

### Surgical procedure and samples

If a clinically relevant ABR was diagnosed, the resorbed bone fragment was surgically removed. For this purpose, the former skin incision was reopened and the residual bone flap was removed. All explanted flaps had been stored in a freezer at − 80 °C until time of implantation.

### Histological processing of the samples

The necrotic bone removed during the revision surgery was sent to the Institute for Osteology and Biomechanics for further examination. The resorbed explanted bone flaps as well as non-transplant controls were processed into undecalcified histological sections and ground sections for microscopic examination. Subsequently, a qualitative and quantitative evaluation of the tissue samples was performed: the presentation of the cell structure as well as the quantification was described using cutting sections. Ground sections were used for the measuring and visualization of the bone structure. Bone volume was used as an equivalent to the porosity of the bone tissue.

Initially, bone fragments were fixed in 3.5% buffered formalin (aqueous formaldehyde solution) for at least 2 weeks until further processing. They were documented photographically and by contact radiography (Faxitron X-Ray Corporation, Wheeling, Illinois, USA).

For the preparation of the tissue samples to cutting and grinding sections the bone was cut into defined sizes with a diamond band saw (EXAKT cutting system Makro 310 CP with EXAKT diamond cutting band 0.3 mm D151 310 segmented, EXAKT Apparatebau, Norderstedt, Germany). Depending on the shape and condition of the removed grafts, the longest possible cross-section of the bone piece with a thickness of approx. 4 mm was sawn out for the grinding sections. A cross-section was also used for the cutting sections, but in a second step, it was segmented into several adjacent bone pieces of max. 20 mm length.

The thin cutting sections were produced in modified form according to Donath using the undecalcified preparation technique for plastic embedding (Technovit 7200, Kulzer Germany) [10, 15]. After dehydration and degreasing of the bone fragments and subsequent polymerization, the polymer blocks with the embedded tissue slices were released from their embedding mold and the ground sections were performed by means of a disc grinding system (EXAKT Mikroschleifsystem 400 CS, EXAKT Apparatebau, Norderstedt).

Following a modification of the Donath protocol, the grinding sections were stained with toluidine blue (N′,N′,2-trimethylphenothiazine-3,7-diamine chloride) as a 1% aqueous solution after immersing in a 10% hydrogen peroxide solution for 5 to 10 min, rinsing with Aqua destillata and drying. The dyeing took effect for 15 min [10].

As described above, the cutting sections were also produced by sawing the samples into sections and subsequent contact radiography. The sections were then stored in formalin and stepwise dehydrated and degreased due to ethanol before polymerization.

Using a microtome (Carl Zeiss AG, Oberkochen, Germany), 4-μm-thin sections were derived and, after drying, stained according to Masson-Goldner, Kossa/Van Gieson, and with toluidine blue.

For this study, the bone volume of the samples was calculated using the Matlab-based statistics and image analysis software MAOSAL, additionally [21]. For this purpose, the established histomorphometric parameter BV/TV (bone volume per tissue volume) was determined [29].

## Results

A total of 13 osseous samples were analyzed. Eleven of these samples were obtained from patients with ABR in whom the resorbed bone had been removed. Two further controls were analyzed. Eight male and 5 female patients were identified with a median age of 43 years (Table 1).

**Table 1:** Clinical characteristics of the patient cohort

Patient no.	Sample no.	Age at time of DC (years)	Sex	Diagnosis leading to DC	Δ*t* DC to reimplantation (months)	Δ*t* DC to explantation (months)
1	E3-14	32	m	TBI	1	15
2	E8-14	44	m	MI	2	12
3	E9-14	26	f	TBI	2	20
4	E10-14	47	f	TBI	4	50
5	E18-14	50	m	SAH	6	23
6	E19-14	43	m	TBI	3	136
7	E20-14	17	f	MI	4	48
8	E9-15	23	m	TBI	2	15
9	H85-16	18	m	TBI	3	14
10	H106-16	57	m	MI	4	18
11	H38-17	5	f	TBI	4	457
12	H10-16	49	m	TBI	-	-
13	H11-16	56	f	TBI	-	-

*Δt*, time interval; *DC*, decompressive craniectomy; *MI*, malignant infarction; *SAH*, subarachnoid hemorrhage; *TBI*, traumatic brain injury

Severe traumatic brain injury was the main cause leading to DC in 9 cases, followed by malignant infarction of the middle cerebral artery in 3 cases and subarachnoid hemorrhage (SAH) in 1 patient. The decision for explantation was made based on clinical findings and imaging, as described in the “Materials and Methods” section. Median time interval between DC and explantation of the resorbing flap was 20 months. The examination of the parameters collected on bone metabolism showed that the mean value of all tested parameters was within the physiological limits (Table 2).

**Table 2:** Endocrinological serum parameters of patients with ABR

Pat. no.	Phosphate (mmol/l)	Protein (g/l)	Albumin (g/l)	Calcium (mmol /l)	TSH (mU/l)	fT3 (pmol/l)	fT4 (pmol/l)	Calci-tonin(ng/l)	Cortisol (μg/l)	Osteo- calcin(μg/l)	25(OH) D3 (μg/l)	1.25(OH)2D3 (ng/l)	b AP (μg/l)	PTH (ng/l)
1	1.18	78.0	42.0	2.32	1.58	5.3	16.0	< 2	134.0	27.3	8.09	36.0	10.90	72.24
2	1.24	73.0	35.0	2.25	0.02	9.7	39.2	< 2	83.0	22.2	29.20	52.0	20.80	61.88
3	1.20	76.0	43.0	2.40	1.76	5.2	14.9	3.0	111.0	18.9	7.66	37.0	11.40	41.16
4	-	-	-	2.17	-	-	-	-	-	-	-	-	-	-
5	1.10	66.0	30.0	2.12	0.45	3.1	15.6	-	163.0	13.2	16.50	34.0	5.50	102.9
6	-	76.0	42.0	2.23	0.57	4.6	16.1	< 2	103.0	15.1	10.50	73.0	4.90	40.74
7	1.04	73.0	40.0	2.33	1.48	4.4	15.6	< 2	150.0	20.7	17.20	41.0	10.10	56.28
8	0.89	75.0	36.0	2.32	2.16	4.3	19.5	-	47	22.6	-	6	15.8	-
9	1.4	69.0	29.0	2.13	3.05	3.3	14.0	3.1	49	32.4	13.75	68.0	17.8	-
10	1.19	-	33.0	2.22	0.86	4	16.9	< 2	85	14.0	17.1	36.0	11.2	-
11	1.36	68.6	33.6	2.43	1.5	4.1	15.8	< 2	142	16.4	28.2	25.0	8	-

*1.25(OH)2D3*, calcitriol; *25(OH)D3*, calcifediol; *b AP*, bone alkaline phosphatase; *fT3*, free triiodothyronine; *fT4*, free thyroxin; *PTH*, parathyroid hormone; *TSH*, thyroid-stimulating hormone

### Qualitative analysis—microscopic analysis of the cutting and grinding sections

**Fig. 2:**
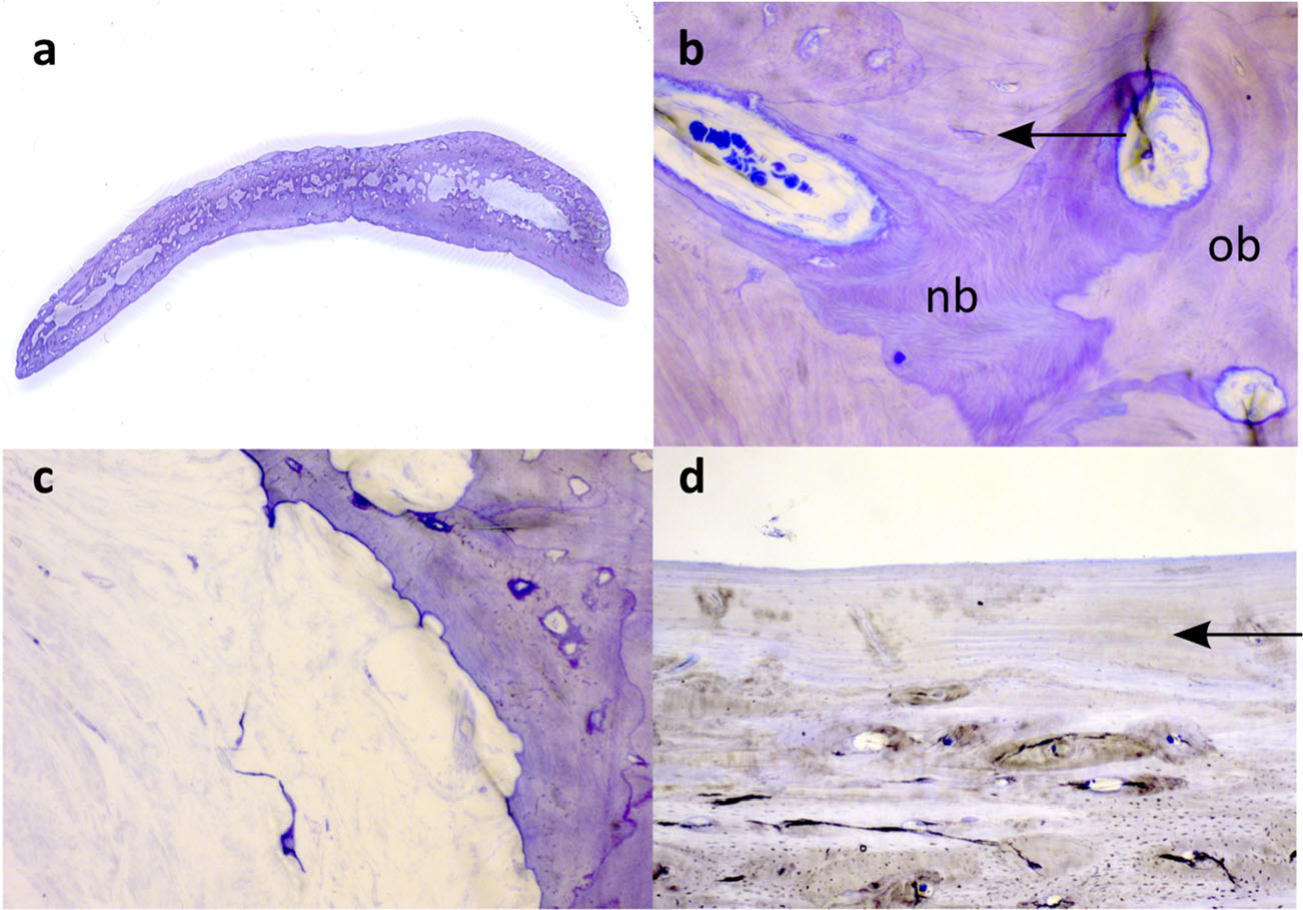
Thin section preparations. **a** Complete overview of sample H85-16. **b** The graft (original bone, ob) contains empty osteocyte cavities (arrow) next to newly formed bone (new bone, nb). **c** Extensive resorption with fibrotic connective tissue in the marrow cavity. **d** Parallel layering of the bone structure, × 25 magnification (toluidine blue; **b**, **c** × 200, **d** × 25)

Already macroscopically, the typical bone structure of cortical and cancellous bone was no longer present in some areas. The graft had thin ends in the marginal areas (Fig. 2a).

Microscopically, a bone remodeling with a build-up of new, vital bone tissue was observed in the entire area of the bone flaps. The remodeling occurred not only from the edge of the transplants, but almost simultaneously in more central sections.

The new bone formations were visible in the form of additions on the original graft bone. In the osteocyte cavities of the new bone formations, the vital cells were clearly visible, whereas no living cells were found in the old graft bone, but empty osteocyte cavities (Fig. 2b). The avital graft bone seemed to serve as guidance for new bone formation by osteoblasts. Bone resorbing osteoclasts were also found.

The remaining marrow spaces were predominantly fibrotic or necrotic (avital). In areas with marrow cavity fibrosis, hardly any new bone tissue was found in the adjacent bone, while there were increased signs of osteoclastic resorption (Fig. 2c).

In general, there was more resorption on the outer side of the transplants facing away from the brain than on the inner side facing the brain. Here, new, elongated, parallel bone lamellae dominated (Fig. 2d).

**Fig. 3:**
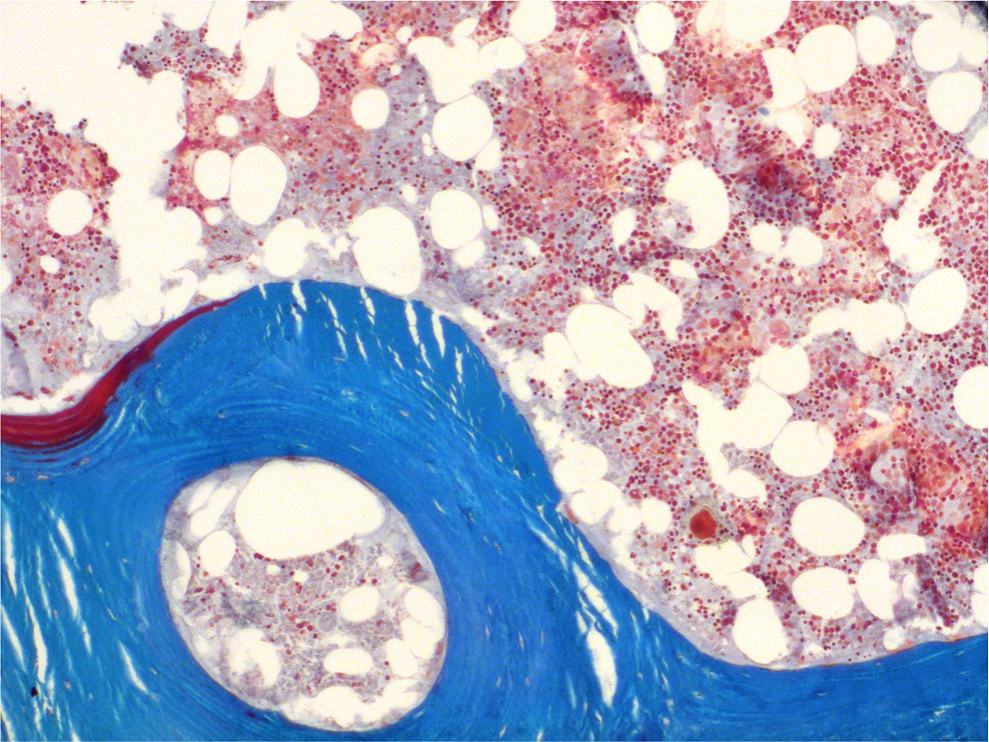
Intact marrow cavity with hematopoiesis of sample H11-16 (Masson-Goldner, × 50)

Taking into account freeze storage, both control samples showed a normal bone structure. The marrow spaces were intact and hematopoiesis activity appeared normal (Fig. 3). Neither control showed signs of fibrosis or necrosis.

### Quantitative analysis—determination of bone volume and bone flap thickness

Following the qualitative analyses, the bone volume was determined in all samples. In the marginal areas of the bone flaps, the two reference samples H10-16 and H11-16 showed values between 70 and 80% BV/TV. With increasing time interval between reimplantation and explantation, the bone volume in the marginal areas of the graft bone increased. Samples with intervals ≤ 20 months were below the values of the reference samples and the samples with intervals of more than 50 months showed higher values.

In a next step, the thickness of the bone flaps was determined. In the marginal areas (MA), thickness ranged from about 3.5 to about 6.5 mm, whereby the measured values lay, with one exception, below those of the controls (Table 3). In the central areas of the samples, the thickness of all bone flaps was also below the reference measurements in the controls, as 87.5% of the measured values were below the mean thickness of the controls. No correlation between time interval between DC and explantation, and bone thickness was found. Due to a lack of serial imaging, however, no statement can be made about the dynamics of osteolysis.

**Table 3:** Results of the thickness measurements in central areas and marginal areas of the bone flap

Sample no.	Δ*t* DC to explantation (months)	Mean bone thickness (mm)		SD (mm)	
		MA	CA	MA	CA
H10-16	0	6.35	5.31	.173	.155
H11-16	0	5.31	5.98	2.965	.096
E8-14	12	5.96	-	1.134	-
H85-16	14	4.68	5.60	1.959	.432
E3-14	15	3.40	-	.294	-
E9-15	15	4.09	6.20	1.158	1.299
H106-16	18	3.83	3.30	1.284	1.716
E9-14	20	5.25	4.38	.420	.206
E18-14	23	3.75	5.56	.955	1.132
E20-14	48	-	1.23	-	.296
E10-14	50	3.70	-	.786	-
E19-14	136	3.01	4.30	.559	.408
H38-17	457	4.39	3.98	2.494	.222

*CA*, central areas; *DC*, decompressive craniectomy; *MA*, marginal areas; *SD*, standard deviation

## Discussion

Compared to the controls, the grafts showed an altered structure, the typical layering of cortical and cancellous bone was mainly eliminated. The measurements showed that the grafts were less thick and had distinctly thinned edges. Histologically, new bone formations were distributed over the entire graft area. We discovered a coexistence of osteoblastic reintegration of autologous bone and necrosis with osteoclastic activity.

The shown resorption processes indicate that the measured thickness decrease of the grafts mainly originates from the outer side as this side showed increased osteoclastic resorption. On the side facing the brain, more elongated parallel bone appositions were found. However, this could not be shown in all samples.

Despite the relatively small control group, the reference values measured in the two control cases for the skull thickness in the peripheral and central areas corresponded to the values in the literature [34].

Fatty bone marrow may be responsible for insufficiency of bone reintegration. We postulate a possible barrier-function of the remaining marrow cavities, since these areas were predominantly fibrotic and showed hardly any adjacent new bone tissue but increased resorption. Thorough rinsing of the implant to remove the remaining bone marrow could be considered. However, a recommendation for this procedure cannot be derived from our study. Further studies to investigate this idea are necessary.

Some risk factors for ABR have already been described in the literature. These include fragmentation of the bone flap, shunt-dependent hydrocephalus, and young age [5, 11, 23, 25].

One option under discussion is the initial implantation of artificial grafts in pediatric patients and fragmented bone flaps, although it should be noted that this involves higher costs which might be of relevance in developing countries [36, 40].

The histological examinations performed in this work provide a new insight into the underlying processes in ABR of autologous cranial grafts.

As endocrinological serum parameters did not differ significantly between patients in whom ABR had occurred and the controls, no systemic serological endocrine predictors could be identified for aseptic bone necrosis in our population.

Since there is no comparable study to date in which results of histological examinations of explanted autologous grafts of the skull have been published, a direct comparison of the own findings with other studies is hardly possible. However, some histological examinations of grafts from other skeletal regions exist. A peripheral bridge-like reintegration starting from the vital bone was observed in homologous transplants from the acetabulum region [27]. In contrast, the skull transplants examined here showed new bone deposits in the entire area, not only in the marginal area of a few millimeters as described for homologous hip transplants.

Both the homologous grafts of the hip and the examined autologous grafts of the skull had been cryopreserved. It is therefore to be discussed whether this alters the marrow cavities in such a way that the body’s own cells are no longer able to colonize these areas, resulting in scar-like fibrosis. Most of the existing studies on the influence of cryopreservation of bone have been performed in animal models [2, 32]. While freezing appears to preserve the morphological state of the mineralized bone tissue and the mineral content, the cellular components are affected more severely. Chan et al. could not detect vital osteoblasts in any of the 18 cryopreserved human bone flaps examined, with a minimum storage time of 4 months in this study [7].

Limitations of this study include the inhomogeneous patient population. The samples were derived from male and female patients of different ages, so that differences in bone metabolism must be suspected. However, endocrinological serum parameters did not differ significantly between patients with ABR and controls. Additionally, the time interval between DC and reimplantation (“freezer time”) varied from 1 to 6 months.

The heterogeneous sample collective and missing samples with time intervals between DC and explantation of less than 1 year do not allow for reliable conclusions about the timing of changes in the transplants. However, the measurements carried out on bone volume indicate that increased resorption of the graft by osteoclasts may occur first, followed by densification of the graft by new bone formation.

It should further be noted that an adverse selection occurred in the samples, as only cases of graft failure could be investigated. With only a limited number of samples, a further statistical evaluation of the measurement results was considered to be of limited use, so that the focus of this work was based on a descriptive approach.

We determined structural changes of the bone as distinct osteopathological features of aseptic bone necrosis. We discovered a coexistence of osteoblastic reintegration and necrosis with osteoclastic activity, demonstrating a disbalance of the complex processes of bone integration. The investigations performed in this study provide new insights into the histological processes in skull grafts after DC and in ABR, allowing further research in the field and providing baseline information for additional studies to expand our knowledge.
